# The Statistics of Insanity, and Idiocy and Cretinism.—Reports of the International Statistical Congress

**Published:** 1856-10-01

**Authors:** 


### Part Second.

### THE STATISTICS OF INSANITY, AND IDIOCY AND CRETINISM. REPORTS OF THE INTERNATIONAL STATISTICAL CONGRESS.

*To the Editor of the Journal of Psychological Medicine.*

Dear Sir,—The subject of statistics as an instrument for the solution of the great problems in mental alienation must often have engaged your attention. To arrive at definite and trustworthy results as to these problems by means of statistics, it is above all things necessary that the field of observation should be as nearly as possible co-extensive with the area of mental alienation. It is, in the next place, important that a similar scheme of observation and record should be adopted at the various stations of that area, so that the different sub-collections of facts may be compared between themselves, and cast up together to constitute homogeneous groups.

That many questions of vast social, political, pathological, and therapeutical importance admit of being settled, or at least elucidated, by an extended and harmonious system of statistical observation carried out in different countries, there can be no doubt. Impressed with this conviction, the International Statistical Congress, held at Paris in September of last year, delegated to sub-committees of the first section the task of framing schemes of statistical record for general adoption. The Reports of M. Parchappe and M. Boudin on Mental Alienation, and on Idiocy and Cretinism—the results of the deliberations of those sub-commitees—were unanimously adopted by the Congress. The Congress was attended by representatives and many distinguished physicians and scientific men from every part of the civilized world. There is hardly any State of importance in which the recommendations of the Congress will not be followed by more or less of practical adoption. It is therefore eminently desirable that England should contribute her share to the common store of knowledge that will be amassed, and in such a form as to admit of comparison and addition with the facts collected in other countries.

I believe that the statistical researches that have hitherto been conducted by the superintendents of our public and some private asylums, constitute a mass of information upon mental alienation not inferior in value to anything of the kind that has been produced in other countries. But it is obvious that methods of observation and tabular forms that differ from each other have been followed, so as often to baffle all attempts at generalization. The numerical records of some public institutions are so meagre as to possess scarcely any value in this point of view. The completeness of our statistical collections is thus marred by two serious defects: one of negligence in observation and record of facts; the other, a want of consistency in the method of record.

But notwithstanding the prevalence of these defects, what M. Parchappe has said is true—namely, that "no other class of diseases has been the subject of statistical studies so general, so persevering." This predilection is to be explained, not so much, it appears to me, by the impulse of humanity and sympathy for the insane, evoked by the genius of Pinel, as by the peculiar nature of the disease itself. Insanity, more than any other disease, leads to segregation: the insane are at once separated from their fellow-men; they are seen apart; and therefore peculiarly lend themselves to statistical study.

This circumstance is at once the explanation of our greater richness in this department of medical statistics, and the justification for further more methodical and extensive investigations.

The schemes of the organization committee, as modified by the sub-committee, and adopted by the Congress, may appear to some to be unnecessarily minute and cumbrous. But those accustomed to rigid analysis of facts—and this process must precede all record of observations in order to make them trustworthy and fitting materials for statistical synthesis—will have learned that no fact is available, or in short *is a fact*, unless it be the result of minute and careful inquiry. There is perhaps no problem in mental insanity that is more likely to be elucidated by extensive statistical study than the multiform one of etiology. But does any one suppose that much light is thrown upon this subject by such vague and meagre information as is afforded in the so-called tables of causes contained in the annual reports of many lunatic asylums? No. These tables obscure the truth, by the interposition of mere words suggestive of false conclusions.

If I were disposed to criticise the programme of the Congress, I should perhaps object that some of the heads classed amongst the "presumed causes of insanity" will in many cases prove to be merely *symptoms*—that is, the early manifestation of an antecedent morbid action or original developmental vice. If, for example, in taking the history of a patient on his admission, and seeking for a circumstance to be recorded as the cause of his disease, we know that obvious mental alienation first appeared after cellular imprisonment, and were to assign that as the "cause," a serious error might be committed. We are too apt to seize upon any salient fact, and accept it as the easy solution of the question. But we might by such a course keep out of sight a long antecedent diseased or defective cerebral condition—a condition which might have been the cause not only of the insanity of the patient, but of the incarceration and the crime of the prisoner. It is this hasty disposition to seize upon the first striking event or prominent feature in the history or character of the patient that vitiates so large a portion of what passes by the name of statistical tables, obscuring, not elucidating, the etiology of insanity, and discrediting the science of statistics.

The reply to this objection would be: 1st, That minute and intelligent inquiry into the history of the patient would in many cases save from this catching at effect for cause—this Atalantan error—and lead to the true primordial pathological condition. 2ndly, That if we guard ourselves against erecting "presumed" causes into "actual" causes, and look upon the salient historical fact only as an early symptom, or a circumstance provocative of early symptoms, we draw no false conclusions, but really add to the stores of useful information.

The objection then really disappears, if we suppose a minute and intelligent analysis of each case; it is a real one only in the records of those whose interrogation of patients is not minute and intelligent.

An objection of more weight would be the apparent exclusion of causes other than those for which a place is provided in the programme. A scheme so *totus teres atque rotundus,* seems to be a declaration that there can be no other causes. This is to anticipate, in part, the very solution which it is the professed object of the application of statistics to discover. Great freedom must be left to each observer to record whatsoever circumstances may appear to him to possess the greatest etiological value. For this reason I am unable to assent to the propriety of the modification of the programme proposed by my learned colleague, M. Parchappe, to substitute for the causes designated under the names *syphilitic diseases, diseases of the skin, fevers,* a group of causes called *different diseases.* If the object of etiological studies be more than the gratification of the love of abstract inquiry—if it be intended to furnish indications for practice, to lead to prevention, the diminution of insanity—then is it in the highest degree desirable to encourage the particularization of the disease that caused the insanity, and to discourage its obscuration under a general phrase which conveys no precise information. For example, it is of immense importance to determine the proportion of cases of mental alienation produced by fever. Weighty hygienic and sanitary measures may be affected. If it be true that fever is a frequent efficient cause of insanity, how powerful the argument for a vigorous sanitary administration! In three Annual Reports under my hand—that of Bethlehem for 1855, the Worcester Asylum for 1855, and of the Crichton Royal Institution for 1855—I find five cases referred to typhus, and one to variola. Had these been confounded under the general phrase of M. Parchappe, valuable truths would have been lost. My own observation in practice has even led me to believe that zymotic diseases occupy a much higher rank in the etiology of insanity than would appear to be justified by existing statistical tables.

For the same reason I would object to a similar confounding under the common name *diseases peculiar to women,* of *slow and difficult development in young girls, suppression of menstruation, puerperal.* It is of the highest importance that the particular disorder of the female sexual system, connected by antecedence with each case of insanity, should be specified. The influence of affections of this class is, in my opinion, so great as to demand the most rigid and penetrating analysis. This branch of the etiology of insanity can only be effectively studied by those whom daily practice brings into close communication with females in all the varied phases of their physiological and pathological vicissitudes. The alienist physician here often sees only the result. The obstetric physician is present at the development of the disease. In time and circumstance he is naturally brought into close approximation with the origin, the etiology. The menstrual function has often a powerful influence over the cerebral action. In many women this influence is so great at every catamenial period, that illusions, hallucinations, and hyperæsthesia, bordering upon mania, arise, and not seldom overpower for a time the reasoning faculties and the will. Where certain diseased conditions of the ovaries and uterus impart a pathological character to the catamenial function, the advent of this period is doubly trying to the mind. The extraordinary self-control which many women are accustomed to exercise under these physical and mental trials, is such that in most instances no external evidence betrays, to ordinary observers, the inward struggle. Now, the characters of the mental disorder associated with paramenial affections are often essentially distinct from those which arise in connexion with pregnancy, puerpery, and lactation. It is therefore important to preserve in asylum-records and lunacy-statistics the precise etiological phenomenon. To confound this in a general expression is to commit the folly of abandoning the very object of statistics—that, namely, of illustrating the causes of disease.

In my observation of suckling women, I have seen that the greatest degree of physical and mental disorder usually comes on towards or after a twelvemonth's lactation—a period which an extended analysis has led me to conclude is the natural term of suckling. I would suggest that when recording the fact of lactation being the apparent cause of insanity, the period of lactation and the number of children to which suck was given be also recorded.

I trust you will forgive me for concluding these remarks by a caution not to expect from the exercise of statistics more than it is capable of telling. It is not so much absolute and isolated facts, as relative and general laws, that statistics can establish. This fundamental truth is often overlooked. One single well-observed case may prove a pathological fact more conclusively than a volume of statistical tables. But, on the other hand, there are numerous laws of the highest practical importance which statistics alone can establish on a firm foundation. Just as Louis, the great medical statist, proved by statistical synthesis and analysis that the relation between fatty liver and phthisis was not an accidental conjunction—which was all that a single dissection would have shown—but a condition intimately dependent upon the nature of phthisis, so statistics alone can prove the relation between epilepsy and insanity, and many other relations of the like kind.

So, again, in regard to the etiology of cretinism and idiocy, no isolated observation can convey a demonstrative proof of the influence of any particular circumstance. Thus, no one could deduce from one or two observations any relation between the absence of iodine in the aliment and cretinism; but MM. Chatin, Grange, Boussaingault, and Fourcault have raised a strong presumption that such relation exists, by showing that iodine exists in the air, water, soil, and alimentary products of most districts; that it exists in considerable proportion in the cereals of the Calvados, where the soil is manured with marine plants; and that the geographical, geological, and chemical media in which iodine was wanting were the countries in which goître and cretinism are endemic. M. Boussaingault has observed that in the Andes, where the inhabitants use a marine iodised salt, they are preserved from cretinism and goître, whilst others who are denied this resource are affected by these diseases. These are facts which the collation of numerous positive and negative observations alone can establish. They will serve for an example of the utility of the inquiries indicated in the programme of the Congress.

I entertain no doubt that the superintendents of our national asylums will gladly lend their aid in augmenting the stores of precise knowledge by adopting more or less of the scheme explained in the following Reports. The schemes actually followed in many of these establishments—and especially that in Bethlehem—already embrace most of the points required. A little extension, some new tables, is all that is necessary.

But who shall collect, compare, and add together the particular statistics of each asylum, workhouse, and department? Is this task—the culminating point whence all great deductions and useful applications must flow—to be left to private enterprise and devotion, or cannot some organisation of a public character be devised to effect it? It is an object worthy of a special committee of the Association of Medical Officers of Asylums; one, indeed, which has already occupied the attention of the Association. The Commissioners in Lunacy might usefully undertake it. The Board of Health might, with the utmost propriety, address itself to this task The Registrar-General's Department, which shrinks before no useful work, whose labours have mainly contributed to the now rapid spread of statistical investigation throughout the world, will, I feel satisfied, undertake it, if not taken up by others.

Believe me, dear sir, yours faithfully,

ROBERT BARNES, M.D., F.S.S.,
Member of the First Section of the
Statistical Congress of Paris.

13, *Devonshire Square*,

*1st Aug.* 1856.

*Scheme for the Statistical Investigation of Mental Alienation, proposed by the Organisation Committee of the International Congress, drawn up by* M. Trébuchet, Reporter.

Does mental alienation make, as several observers assert, rapid progress? Is it true that our political revolutions, in some kind periodical—our industrial crises, our exchange gambling, the feverish agitation caused by the loosening of the spirit of speculation, that panting race after fortune which specially characterises the present generation;—is it true, we ask, that these different circumstances exert a deep and growing perturbation in our intellects? Certainly, the question is well worthy of being examined. It would be, in truth, a dark cloud in the brilliant picture of the actual conquests of the human mind in the path of material interests, this rapid development, if proved, of the most terrible of maladies.

This question statistics alone can solve. Statistics alone can teach us if the proportion of the insane to the population tends to increase; if, supposing this proportion stationary, the nature, character, intensity of this cruel affection undergo modifications in any direction; lastly, if, thanks to the advance of curative methods, society—families behold, year by year, a greater number of these sad exiles from human reason return into their bosom.

The position of mental alienation may be determined by two distinct operations: 1st, by the periodical enumerations, of which the population is the object in almost every State; 2nd, by the annual reports of public and private lunatic asylums.

The two methods must be employed simultaneously, for each has its advantages. The census, in fact, supposing it exact, makes known the total number of insane treated both at home and in special establishments. The reports of these establishments, if they give only the position of alienation, make known the annual movement, numerous and varied information, which it is not possible to obtain in the course of a census.

The programme of questions must, therefore, vary, according as the one or the other method of observation is employed.

### TABLES.

### I.—Questions to be stated in the Census.

The insane must first be classed in two great categories, comprising—one, the insane treated in special establishments; the other, the insane treated at home.

The following is the minimum of questions to be put for the insane of both classes:—

A.—*Insane, properly so called.*

Number.
Sex.
Age.
Profession.
Presumed causes of the alienation.
Degree of instruction before the disease.

B.—*Idiots or Cretins.*

Number.
Age.
Sex.
Number of cases in which idiocy was or was not congenital. [In case of non-congenitality, learn at what age the disease became manifest, and what circumstances, general or local, may have determined or favoured its development.]

Topographical situation of the places where idiocy prevails. [Plains, valleys, mountains.]

Profession, and degree of comfort of the parents.

C.—*Senile Dementia.*

Sex.
Age.
Profession.

II.—Questions concerning Alienation treated in Special Establishments.

A.—*Administrative Details.*

Number of establishments—public (at cost of State, province, community).
" " private.

Analysis of legislation affecting both kinds of establishments, chiefly as regards—1st, the public security, 2nd, individual liberty.

B.—*Movement* (*Admissions and Discharges*).

Number, by sex (for the last two years), of insane remaining in the establishments on the 31st December of each year.

Number admitted in each year—for first time (by sex).
" " for relapse (by sex).

[Indicate the number of relapses for each sex, according as they have occurred in the first, second, third, and so on to the fifteenth year of cure.]

Number in each year:—
1st. Of discharged—for cure (by sex).
" " for other causes (by sex).
2nd. Of dead—from natural causes (by sex).
" from accident (by sex).
" from suicide (by sex).

Total number of days of sojourn in the year (by sex).

Out of the whole number treated each year, how many were reported . . . . . . . { curable (by sex). incurable (by sex).

C.—*Different Details concerning the Admitted of each year.*

1. *Ages at the time of Admission.*

The classification by age may be established thus:—From 0 to 15 years; from 15 to 20; every 5 years, up to 40; every 10 years, up to 100.

In each category of age the number of insane must be given by sex, and for each sex by civil state (single, married, widowed).

2. *Professions* (*by Sex*).

1. Liberal professions:—
   Ecclesiastics.
   Jurists.
   Physicians, surgeons, apothecaries, midwives.
   Professors and literary men.
   Public functionaries.
   Employés.
   Artists (painters, sculptors, musicians, &c.)
2. Soldiers and sailors.
3. Renters and proprietors (living on their means.)
4. Industrial and commercial professions:—
   Manufacturers and artisans.
   Merchants and dealers wholesale.
   Merchants in retail.

5. Manual or mechanical professions:—
   - Workmen:
     - Miners.
     - in metals.
     - in wood.
     - weaving.
     - in building.
     - in leather and skins.
     - in dyeing.
     - in articles of dress, hairdressing, and boots and shoes.
     - Others.
6. Agricultural professions:—
   - Proprietary cultivators.
   - Agricultural labourers (farm-servants, shepherds, wood-cutters, &c.
7. Workpeople on wages (domestic servants, clerks, journeymen).
8. Other professions.
9. Without professions.
10. Unknown professions.

3. *Presumed Causes of Insanity* (*by Sex*).

1. Physical:—
   - Hereditary.
   - Effects of age (senile dementia).
   - Effects of labour { intellectual. / manual.
   - Habitual irritability.
   - Want and misery.
   - Onanism.
   - Venereal abuses.
   - Syphilitic diseases.
   - Diseases of skin.
   - Epilepsy, convulsions.
   - Violent emotions, shocks, fright.
   - Fevers.
   - Slow and difficult formation (in young girls).
   - Accidental or definitive suppression of menstruation.
   - Puerperal.
   - Blows and wounds.
   - Central concussions, &c.
   - Hydrocephalus.
   - Cephalalgia.
   - Cerebral congestion.
   - Apoplexy, paralysis (consequences of).
   - Other physical causes.
2. Moral:—
   - Grief resulting { from loss of fortune. / from loss of cherished person. / from disappointed ambition.
   - Love.
   - Jealousy.
   - Pride.
   - Political events.
   - Sudden passage from active live to an inactive one, or *vice versâ*.
   - Isolation and solitude.
   - Imprisonment { simple. / cellular.
   - Nostalgia.

Religious sentiments carried to excess.
Contact and assiduous intercourse with the insane.
Other moral causes.

3. Unknown causes.

4. *Months of Admission.*

Indicate for each month the number of admissions by sex.

5. *Number, by Sex, of Insane originating* { from towns. / from country.

6. *Aggravated Circumstances of the Disease.*

Number, by sex, of insane affected { with paralysis. / „ epilepsy.

7. *Duration of the Treatment*—1*st, of Insane cured; 2nd, of Insane dead* (*by Sex*).

8. *Cures and Deaths* (*by Months*).

Give, for each sex, the number, by months, of cures and deaths.

9. *Age, by Sex, of Insane cured and dead, in the Month of Cure and of Death.*

The classification indicated for the ages at time of admission may be adopted.

10. *Cures and Deaths, by Sex, according to Professions.*

Reproduce the classification adopted for admissions.

11. *Curative Methods.*

Describe the curative method employed in each establishment.

12. *Occupations of the Insane.*

Indicate the principal works in which the insane, divided by sex, have been occupied.

*Information concerning Cretins or Idiots.*

Numbers, by sexes, remaining on the 31st of December of each year.
Number admitted each year, by sex and age.
Number, by sex, of cases { congenital. / non-congenital.
Number, by sex, of the cretins' or idiots' offspring { of towns. / of country.
(Add information as to topographical and other conditions of the localities to which the greatest number of cretins and idiots belong.)
Profession, and, as far as possible, the degree of comfort of parents.
Number, by sex, of cretins or idiots discharged in the year { cured. / for other reasons.
Number, by sex, of cretins or idiots died during the year { from natural causes. / „ accidents.
Indicate the principal curative methods.
Indicate the employments of the cretins or idiots of each sex.

---

*Report on the Statistics of Mental Alienation. By Dr.* PARCHAPPE, *General Inspector of the First Class of Lunatic Asylums and Prisons.*

[This Report is the result of the deliberations of the Committee on Mental Alienation of the First Section of the Congress, consisting of—Dr. Parchappe,

President; MM. Trébuchet, Hubertz, Vingtrinier; Drs. Barnes, Virchow, Poisson, Greenhill, Boudin, Bertini, Villermé, Meding, Tholozan.]

The statistics of mental diseases have assumed for many years a great development both in extent of research, and in the importance of the publications bearing upon the subject.

The records of science possess at this moment a rich collection of documents, the results of studies upon mental alienation undertaken either by statisticians, alienists, or public administrations, in the principal States of Europe and America.

No other class of diseases has been the subject of statistical studies so general, so persevering. It is not without interest to explain, to justify this kind of predilection in statistics for mental alienation.

Towards the end of the eighteenth century, the sudden revelation of the sufferings imposed from time immemorial upon the insane in prisons, houses of correction, and even in hospitals, awoke, in France, in England, in Germany, a deep and lasting sympathy, which soon spread throughout all civilized countries.

Under the impulse, continued to the present day, of an immense concourse of benefactors of humanity, who were personified in the beginning under the venerated names of Pinel, William Tuke, and Langermann, a reform, medical, legislative, and administrative, was proposed and undertaken in all that relates to the insane, the complete realization of which will be one of the glories of the nineteenth century. Is it necessary to indicate the importance of the part which belonged to statistics in this vast and difficult enterprise? Is it not enough to say that without exact statistical data, the organization of the public charity by the means the most efficacious and the most important—the creation of asylums—cannot achieve its end with certainty and success, and is exposed to encounter, in feeble trials, only irreparable errors and ruinous deceptions?

But at the same time that the necessity of resorting to statistics for the determination of the administrative programmes was admitted, there arose, out of the very researches attempted in order to attain this end, the fundamental question of the relation of the number of the insane to the population, including the appreciation of the variability of this relation according to time and place. Then, by a logical consequence, there came the capital question as to the influence of the degree of civilization on the development of mental alienation, entailing, as means of solution, all the most delicate and the most difficult secondary questions of etiology. In the midst of this movement of the realization of benevolent institutions destined to make amends to the insane for the wrongs of a suffering past, and in proportion as the applications became multiplied and spread into different countries, Medicine naturally felt itself called upon to justify the promises of amelioration in the condition of the insane which had been held out as motives in the programme of reform.

Medicine invoked the aid of statistics to prove the efficacy of the curative treatment by the number of cures, and to show, by the diminution of the mortality, and by the advantages of the organization of labour in the public asylums, the happy effects of the palliative treatment. The long and serious discussions raised by the problem of penitentiary reform led to the question of the influence of cellular imprisonment in the production of mental alienation. The last word in this question can only be spoken by statistics, to which it belongs, also, to demonstrate the necessity for the creation of special institutions for the criminal insane.

But in proportion as benevolent institutions and methods of medical treatment were being developed to the advantage of the insane, the inadequacy of their resources soon became manifest as regards two great classes of unhappy beings, who, destined irretrievably, by the misfortune of their birth, to a condition still more wretched than that of the accidental victims of insanity, found themselves necessarily included in the sphere that reform was called upon to embrace, but in which, in fact, for too long a time they remained neglected.

In relieving and treating the insane, it was impossible to avoid being met by the idiots and cretins.

In the presence of these two great misfortunes, the administration and medicine have not misunderstood their duty; and at the very outset they called upon statistics to prepare a better future, by comprising in a special manner in its luminous and fruitful investigations the double question of idiocy and cretinism.

Studies investigated by interests so powerful could not remain sterile. A great number of questions, perhaps the most important, must be considered as settled, at least, of those which concern mental alienation properly so called.

Thus statistics have shown that insanity is curable, and that more than one-third of the unhappy beings who, from whatever cause, seek for the aid of medical treatment in well-ordered establishments, are cured.

And even, without the positive information furnished by statistics, a simple visit to one of these establishments proves beyond reply that what has been realized for the welfare of the incurables has surpassed the promises of science and the hopes of charity.

Data more or less approaching to exactitude have been obtained in several countries as to the proportion of insane to the population.

The influence exerted, as predisposition, on the development of insanity, by sex, age, climate, seasons, civil condition, professions, has been appreciated.

Facts have established that the agglomeration of population in large towns favours the development of insanity, which is, on the contrary, restrained by their dissemination in rural districts.

In determining the whole force of heredity in the etiology of insanity, statistics have limited its action to an influence of predisposition, and refused to attribute to it the characters of a necessarily determining cause.

The study of the determining causes of mental alienation, properly so called, has led to the recognition of the predominance of moral causes over all the other causes, and has revealed a happy agreement between the demonstrations of statistics and the teachings of morality. In fact, a profound study of the etiology of insanity permits us to affirm, that the best means of preserving oneself from a disease, of which the most distressing character is to rob man of his most precious prerogative, the use of reason, consists, for all of us, in impressing upon our lives the direction conformable to the rules of morality—that is to say, moderation in the satisfaction of all the legitimate tendencies of our nature, and the subordination of all those tendencies to the supreme end of human life, the never-dying aspirations after moral perfection. But notwithstanding the wide bearing, and the certainty of the teachings hitherto obtained, it is evident, that even for those which rest the most firmly upon facts, the sanction of large numbers is still indispensable.

Moreover, we cannot conceal from ourselves that contradictions have frequently presented themselves in facts and interpretations, and that out of a given number of points, observations are insufficient, or even entirely wanting.

Lastly, the solution of some of the most important questions supposes a generalization of observation by numerical facts which shall embrace all the conditions of time and place, and consequently comprehend the statistical study now continued for a long time in many countries.

To this necessity for the generalization of statistical studies for the elucidation of general problems, is attached the necessity of instituting methods of observation, whence facts exactly comparable may result.

It is already long since the utility has been insisted upon of harmonizing, for the purpose of solving universal questions, the particular statistical studies that may be undertaken by isolated *savants*, and, *à fortiori*, the general studies which embrace a whole country, and which hardly any but public administrations are able to realize.

The transformation into fact of this general tendency of the mind to the rapid and complete perfection of statistics, is the ruling thought of the International Congress; it is also the end of which we all follow with our wishes and our efforts the near and sure realization.

This end has been generally attained, in as far as regards mental alienation, by the question put forth by the third French sub-committee, and printed in the programme, pp. 111 to 116.

The modifications which the examination of this document has led us to regard as useful, and which I shall have the honour to submit, in the name of the First Section, to the approbation of the Congress, bear only, notwithstanding their real importance, on details; they do not change in any way the principle of the work, nor even the realization of this principle; they are calculated only to perfect it, and to render it in every way acceptable to all.

*Modifications in the Interrogation relative to the Statistics of Mental Alienation.*

1. Add senile dementia to alienation properly so called, and separate into two classes idiots and cretins.

A. Insane.
B. Idiots.
C. Cretins.

2. Substitute, in what relates to idiots, for the paragraph beginning with the words "Number of cases," &c., and ending "its development," the indications of the etiological particulars adopted for cretinism, and not to omit among the predisposing causes, *heredity*.

3. A. To the administrative particulars add—
  1. The mean cost of the place of a pauper-lunatic in public institutions.
  2. The mean cost, *per diem*, for the support of a pauper lunatic in public institutions.

4. B. *Movement*. Last paragraph, substitute the word "admitted" for "treated."

5. C. *Sundry information*. 3. *Presumed causes of alienation*.

Separate from the table of causes under the special name of predispositions, or predisposing causes, heredity.

*Physical Causes*.

Erase the causes designated under the names *habitual irritability*, *excess of manual labour*.

Substitute for the causes designated under the names *syphilitic diseases*, *diseases of the skin*, *fevers*, a group of causes called *different diseases*.

Substitute for the causes designated under the names *convulsions*, *hydrocephalus*, *cephalalgia*, *cerebral congestion*, *apoplexy*, *paralysis*, &c., a group of causes designated under the name of *diseases of the nervous system*.

Substitute for the causes designated under the names *slow and difficult development in young girls*, accidental *or definitive suppression of menstruation*, *puerperal*, a group of causes called *diseases peculiar to women*.

Unite in a single group of two causes *onanism* and *venereal abuses*.

Unite in a single group of causes under the names *blows*, *falls*, *wounds*, &c., the two causes designated under the names *blows and wounds*, *cerebral shocks*, &c.

Remove into the tables of moral causes, 1. the cause, *excess of intellectual labour*, under the same name; 2. the cause, *violent emotions, shocks, fright*, under the name *fright*.

*Moral Causes.*

Add the following causes: *domestic grief, remorse, anger, joy, wounded modesty*.

Strike out the cause designated under the words *contact with and visiting the insane*.

6. *Aggravative circumstances of the disease.*

Make all statistical studies bear distinctly upon *alienation with epilepsy*, and *alienation with general paralysis*, which constitute, in relation to alienation properly so called, two species widely distinct from simple mental alienation.

6. There would be reason to admit, as a distinct species, *mental alienation with pellagra*, at least for Italy, if not for France, where it is sometimes observed in some southern departments; and then it would be necessary to give a prominent place amongst the physical causes to *pellagra*.

7. It is important to exclude from the scheme of statistical studies on mental alienation, *delirium tremens*, which is often the cause of sequestration in lunatic asylums. If it were admitted, it would be necessary to bring the whole range of statistical studies to bear separately upon this disease

8. Lastly, it is necessary to ask, in reference to dead lunatics, a table of *the causes of death*, made to correspond with the nomenclature adopted for deaths in the general population.

---

### *Report on Idiocy and Cretinism. By* M. Boudin, *Reporter.*

Gentlemen,—You have heard, in your sitting yesterday, the report of M. Parchappe on alienation properly so called, and senile dementia. The programme of the Congress had made of idiocy and cretinism a simple paragraph of the question of mental alienation.

Considering the high importance of these two infirmities, and the numerous characters which separate them from alienation proper, your First Section has deemed it fitting to devote to them a special report. This decision is further justified by the great works of which idiocy and cretinism have within the last two years been the object amongst many learned men, and several States of Europe.

Amongst these works we will specially mention—

1. In Norway, the researches of Doctor Stolst, published in Christiania in 1851.

2. In Denmark, the statistical studies of our colleague, M. Hübertz, published at Copenhagen in 1851.

3. In England, an important work by Dr. Stark on mental alienation and idiocy in England, Scotland, and Ireland, published in 1851, in the 14th volume of the "Journal of the Statistical Society of London."

4. In Germany, the works of MM. Falk, Escherich, Roesch, and Maffei.

5. In Italy, the great Report of the Commission created by the King of Sardinia for the study of Cretinism (Turin, 1850); the Statistics of the Kingdom, published in 1851.

6. Lastly, in France, the works, of MM. Grange, Tourdes, Chatin, Niepce, Bouchardat, Baillarger, &c.; works, the publication of which scarcely dates beyond the last two or three years.

Let us add, that in France the Government has associated itself with this great scientific movement: the Minister of War, by indicating, in his returns of the census of the army, the number of exemptions on account of idiocy, cretinism, and imbecility, from the year 1850; the Minister of Commerce, by entering resolutely, at the time of the census of 1851, into the broad and fertile path of numbering the *apparent infirmities*.

Such a mass of recent works, undertaken at one time in so many different points of Europe, both by learned men and Governments, testifies sufficiently to the importance everywhere attached to the study of cretinism and idiocy, at the same time that it justifies your first section in having determined to devote a special report to these two infirmities.

It is by his intelligence that man is distinguished from the brute. Man is man only when he enjoys the fulness of his intellectual faculties. The partial or total loss of those noble faculties degrades him, excludes him in some sort from his order: it makes of him a burden for society, often even a danger.

For this reason, the Organization Committee of the Congress was happily inspired when it comprised in its programme the statistical investigations to be undertaken on mental alienation in general, and on idiocy and cretinism in particular.

Even as, for the physician, the determination of the disease, its nature and intensity, precedes all therapeutical operations, so also in presence of a social evil the first duty of an administration consists in ascertaining the number, the rank, and the source of the victims.

In this respect, statistics constitute the first step—a necessary step—towards the research of the means which may ultimately be opposed to the evil.

What is, at the present day, the number of idiots and cretins?

Does this number follow an increasing rate, or a falling one?

What is the proportion of these two kinds of infirmities in each of the two sexes, and in the different ages of life?

In what proportion do these two infirmities share, annually, to the number of exemptions from military service?

Is it true, as it has been said, that in proportion as cretinism dwells in certain localities, the number of idiots augments there?

Are cretinism and idiocy hereditary?

What is the danger of marriage between cretins?

What are the topographical and meteorological conditions which favour the development of these two infirmities?

Are there any means of combating idiocy and cretinism efficiently, and what are these means?

These, gentlemen, are some of the different questions raised by the study of the infirmities which it is our duty to examine. It is enough to formulate them, in order to make evident all the medical weight, and to give a just idea of the importance of a good enumeration of idiots and cretins. Amongst other useful indications, this enumeration, if correctly made, will demonstrate the increasing or decreasing progress of the two diseases, the epoch of their first manifestation in the country; it will enable us to verify what there is of truth in the assertion according to which, in certain localities, the number of idiots has increased in proportion as cretinism has diminished.

To illustrate with a clear light these different questions, a good enumeration of idiocy and cretinism must comprise the absolute and proportional number, the sex, age, civil condition, and profession of the individuals.

It must mention particularly the individuals of the male sex of twenty years of age, as this indication furnishes the measure of the share which each infirmity takes in the diminution of the recruitable population; and on the other hand, the proportion of the number of infirm serving to establish the existence or non-existence of endemicity.

It must indicate the age at which the infirmity began to be manifested; this datum possessing the advantage of fixing science upon the congenital or non-congenital nature of the two affections.

It must divide the infirm, as much as possible, according to the degree of the evil.

In this respect cretins may be classified into Cretins, Half-cretins, and Cretinous.

It must indicate the aptitudes and habitual occupations of the individuals.

The complications must be the object of special attention. In this respect we ask for particular researches on epilepsy, blindness, deaf-dumbness, scrofula, and lastly, on the presence or absence of goître.

The special diseases of cretins, and if cause exist, their pathological immunities, must be indicated.

It is proper to determine the number of deaths of cretins; the age of the deceased; lastly, the diseases which have been the cause of death.

The number of marriages between cretins must be determined with care; and special attention will be given to the determination of the fecundity, and above all, to the heredity of the infirmity.

It is important to denote the attempts undertaken with the view to combat the evil, and to ascertain what results have been observed.

*Researches relating to the Parents.*

As to the parents of idiots and cretins, it is proper to note with care their race and nationality, the degree of comfort, and profession.

In regard to race, you all know that M. Humboldt has remarked the immunity of the red-skins against goître. This is not all: about twenty-four years ago, a French medical society offered for *concours* the following question:—

"Why is the Jewish woman exempt from goître?"

If it were established that there are immunities of races against goître, would it be impossible for something analogous to exist for cretinism?

As for the grave question of heredity, we must inquire if the individuals are born of—

Father idiotical or cretin;
Mother idiotical or cretin;
Father and mother idiotical or cretin.

*Topographical Researches.*

Idiots and cretins should be enumerated separately in towns and in the country.

The statistics should define with care the geographical position of the localities enumerated; and special attention should be given to the hypsometric data.

It has been said that the endemic territory of cretinism does not rise in

Switzerland . . above 1000 mètres;
Piedmont . . . above 2000 „
South America above 4700 „

All these assertions, however respectable their source, want to be verified.

The soil must be studied as to its configuration, its geological nature, chemical composition; lastly, as to the kind of cultivation.

The drinkable waters must be examined in the double point of view of their temperature and chemical composition.

Amongst the meteorological agents, special attention must be paid to temperature, light, moisture.

Lastly, Gentlemen—and this is an observation that applies to every statistical publication, as well as to every scientific labour—it is important to indicate the method pursued in the collection of the facts, and the quality of the persons to whom this collection has been entrusted. It is easily understood, for example, that in a study of a question of medical appreciation, the facts will have so much the more weight, as competent physicians shall have taken a greater share in the collection.

From these considerations, we have the honour to propose to the Congress to adopt the programme modified as follows:—

*a.* Substitute the words "idiots or cretins" by "idiots and cretins."

*b.* Place at the head of every document a short description of the method of enumeration followed.

*c.* Preserve the terms of the programme concerning the number, sex, and age of the individuals, as well as the profession and the degree of comfort of the parents; only, to add the race and nationality of the parents.

*d.* Preserve the paragraph relating to the congenital origin of the infirmity.

*e.* Complete the paragraph "*Topographical Situation*" with the following words: denote the altitude and aspect of the places enumerated, the configuration and geological nature of the soil; indicate the chemical composition and the temperature of the drinkable waters.

*f.* Indicate the absolute number, and the proportional number to population, of idiots and cretins—1st, in towns; 2nd, in country.

*g.* For the chief centres of endemicity, indicate both the number of individuals and the total of the population.

*h.* Indicate the civil state of the infirm, and the number of marriages between cretins.

As to the interrogation of the individuals admitted into special establishments, the Section adopts the programme with the following additions:—

1. Indicate of the treatment directed against the infirmity itself.
2. Indicate the principal particular complications—epilepsy, deaf-dumbness, scrofula, goître.
3. Indicate the principal diseases for which idiots and cretins have been admitted: state, if there be occasion, the pathological immunities.
4. Indicate if the idiots and cretins are born of—

A father idiotical or cretin;
A mother idiotical or cretin;
Father and mother idiotical or cretin;
Parents affected with mental alienation proper.

Such is, Gentlemen, the programme which, in the name of your First Section, we have the honour to submit for the approval of the Congress.

